# Comparative Evaluation of the Effectiveness of Laser-Assisted Irrigation and Ultrasonic Irrigation on Postoperative Pain in Single-Visit Endodontics: A Systematic Review

**DOI:** 10.7759/cureus.84947

**Published:** 2025-05-28

**Authors:** Arti Gulhane, Kishor D Sapkale, Abrar Sayed, Manoj Ramugade, Shamal Kamble, Aditi Magar

**Affiliations:** 1 Conservative Dentistry and Endodontics, Government Dental College and Hospital, Mumbai, IND

**Keywords:** laser-activated irrigation, postoperative pain, root canal disinfection, single-visit endodontics, ultrasonic irrigation

## Abstract

Effective irrigation plays a critical role in the success of root canal therapy by eliminating residual microbes and debris from inaccessible canal regions. Advanced irrigation techniques have been developed over the years that activate the irrigant leading to increased canal penetration and reduced post-treatment discomfort. These include activation by light, vibration, heat, laser, sonic and ultrasonic methods. The present systematic review compared the effectiveness of laser-activated irrigation (LAI) and ultrasonic irrigation (UI) in reducing postoperative pain following single-visit endodontic treatment. A comprehensive search of electronic databases and grey literature from January 2000 to April 2024 was conducted. Nine randomized controlled trials involving 547 patients met the inclusion criteria. The studies were conducted across four countries and assessed various activation techniques including diode laser, Er:YAG, shock wave-enhanced emission photoacoustic streaming, photon-induced photoacoustic streaming, passive UI, and continuous UI. LAI demonstrated faster pain reduction in the first 24 to 48 hours post-treatment, while UI showed comparable efficacy beyond this period. Both methods were significantly more effective than conventional syringe irrigation. No severe adverse events were reported. The overall risk of bias was found to be low in eight studies, with one study showing some concerns. The results suggest that both LAI and UI are effective in minimizing postoperative pain, with LAI being more effective for early pain control. Integration of either technique is recommended in clinical endodontic protocols to enhance patient comfort and treatment outcomes.

## Introduction and background

The success of root canal treatment (RCT) relies heavily on the thorough disinfection and decontamination of the root canal system to eliminate microbial biofilms, necrotic tissue, and debris. Inadequate cleaning can lead to persistent infections, postoperative pain, and treatment failure [1]. Mechanical instrumentation alone is insufficient to achieve complete debridement, particularly in areas such as lateral canals, isthmuses, and dentinal tubules. These structures are too narrow for instruments to access, allowing residual bacteria and necrotic tissue to remain. As a result, irrigation by chemical agents complements the mechanical preparation by enhancing microbial elimination, dissolving organic matter, and flushing out debris from inaccessible areas within the canal system [2].

Traditionally, syringe-based irrigation with solutions such as sodium hypochlorite (NaOCl), ethylenediaminetetraacetic acid (EDTA), and chlorhexidine (CHX) has been widely used in endodontic practice [3]. However, the conventional static irrigation technique by syringes has significant limitations, including limited penetration of irrigants into the complex root canal anatomy, inadequate removal of smear layers, and ineffective disruption of bacterial biofilms. Furthermore, excessive apical extrusion of irrigants can lead to inflammation, tissue damage, and increased postoperative pain. Therefore, advanced irrigation techniques have been developed over the years that activate the irrigant leading to increased canal penetration and reduced post-treatment discomfort [4]. These include activation by light, vibration, heat, laser, sonic and ultrasonic methods [4,5].

Laser-activated irrigation (LAI) uses high-energy light pulses from erbium lasers, such as Er:YAG and Er,Cr:YSGG, to activate the cleaning solution inside the root canal. These laser pulses create tiny shock waves and bubbles (a process known as photoacoustic streaming and cavitation) that rapidly move the irrigant throughout the canal system. This dynamic fluid motion helps to remove the smear layer, push disinfectants deeper into the tooth’s fine structures, and break up bacterial biofilms more effectively than conventional methods [6]. The cavitation-induced hydrodynamic forces generated by LAI allow for superior debris clearance in irregular canal morphology which contributes to reduced postoperative pain by decreasing the inflammatory burden. The advantages of LAI include minimal apical extrusion, superior debris clearance in complex canal anatomy, and enhanced penetration of irrigants into dentinal tubules [7].

Photon-induced photoacoustic streaming (PIPS) and shock wave-enhanced emission photoacoustic streaming (SWEEPS) are advanced laser-activated irrigation techniques that have significantly improved the efficacy of root canal disinfection [8,9]. PIPS utilizes low-energy, short-pulsed erbium lasers (such as Er:YAG) to create photoacoustic shockwaves within the irrigant. These shockwaves induce vigorous streaming and cavitation without requiring the fiber tip to enter the root canal, thus reducing the risk of apical extrusion [8,10]. In contrast, SWEEPS is an enhancement of PIPS that employs paired ultra-short laser pulses to generate synchronized shock waves within the irrigant. This results in intensified fluid agitation and deeper penetration of the irrigant into lateral canals and dentinal tubules [11].

Ultrasonic irrigation (UI) utilizes high-frequency ultrasonic waves (25-30 kHz) to activate the irrigant within the root canal system. It has demonstrated superior efficacy in removing smear layers and bacterial colonies, particularly in curved canals and anatomical complexities [12]. It generates acoustic streaming and micro cavitation, leading to effective disinfection, disruption of bacterial biofilms, and enhanced removal of debris from canal irregularities [13]. The mechanical streaming effect of UI ensures improved contact of disinfecting solutions with root canal walls, leading to better microbial control. However, concerns regarding the apical extrusion of irrigants and potential irritation of periapical tissues have been raised, necessitating an evaluation of its impact on postoperative pain [14].

Despite the proven advantages of LAI and UI in enhancing root canal disinfection, their impact on postoperative pain remains an area of ongoing investigation [15-18]. Postoperative pain in endodontics can result from inflammatory responses, residual bacterial presence, or apical extrusion of debris and irrigants [19]. Over the years, evidence has demonstrated that LAI may reduce pain due to its enhanced antibacterial effects and minimal mechanical stress [20]. Likewise, UI has also been demonstrated to lower pain levels through improved decontamination due to continuous irrigant activation [21]. There is a need to analyze which method is more effective in reducing post-endodontic pain while taking into consideration the differences in study designs, laser and ultrasonic parameters, and pain assessment scales.

Single-visit root canal treatment has become increasingly common in contemporary endodontic practice due to its clinical efficiency, reduced patient visits, and comparable outcomes to multi-visit procedures. It is especially indicated in cases involving vital pulp tissue, controlled symptomatic irreversible pulpitis, or non-complicated necrotic cases without acute infection [22]. For patients with time constraints, dental anxiety, or logistical difficulties in attending multiple appointments, single-visit endodontics offers a practical and patient-centered solution. Additionally, with advancements in disinfection techniques, including laser and ultrasonic activation, adequate canal debridement and microbial control can be reliably achieved within a single appointment [23]. This approach not only minimizes the risk of inter-appointment contamination but also enhances patient comfort and compliance, all of which are directly related to postoperative pain.

In this context, the present systematic review aims to critically evaluate and compare the effectiveness of LAI and UI in reducing postoperative pain in single-visit endodontic treatment. The findings would provide clinicians with a decision on the subject concerning which method is a more effective irrigation activation system for reducing postoperative pain following single-visit endodontic treatment.

## Review

Methodology

Protocol Development

This systematic review was conducted following the Preferred Reporting Items for Systematic Reviews and Meta-Analyses (PRISMA) 2020 guidelines to ensure transparency and standardization of the methodology [24]. The protocol was prospectively registered in the PROSPERO database under the registration number CRD42024543644.

Research Question

The research question was formulated using the Population (P), Intervention (I), Comparison (C), and Outcome (O) (PICO) framework to ensure a structured approach. The study aimed to determine whether there is a difference in the effectiveness of laser-activated irrigation and ultrasonic irrigation in reducing postoperative pain in single-visit endodontic treatment. The population included patients with necrotic pulp or irreversible pulpitis, and the intervention involved root canal irrigation. The comparison was made between ultrasonic irrigation and laser-activated irrigation, with the primary outcome measure being the extent of postoperative pain reduction. The review included randomized and non-randomized clinical trials, while cross-sectional studies, in vitro studies, animal-based studies, review articles, letters to the editor, technical notes, case reports, and case series were excluded.

Eligibility Criteria

Studies were included if they investigated patients between the ages of 18 and 65 years, regardless of gender, undergoing root canal treatment. Only articles comparing the effectiveness of laser-activated irrigation with ultrasonic irrigation and specifically reporting postoperative pain reduction were considered. Patients included in the studies were required to have a non-contributory medical history to avoid confounding factors.

Studies were excluded if they involved teeth with developmental anomalies, calcifications, internal or external resorption, or fractures. Similarly, cases with a history of previous endodontic treatment, immature teeth with open apices, or the presence of acute pus discharge were omitted. Additional exclusions were made for studies involving pregnant or lactating women and individuals with mental health conditions that could impact treatment compliance and pain perception.

Search Strategy

A comprehensive electronic search was conducted for studies published between January 2000 and April 2024 using databases such as PubMed, SCOPUS, EMBASE, Google Scholar, and EBSCOhost. The search strategy was structured to maximize the retrieval of relevant literature, including gray literature sources such as Google Scholar, Greylist, and OpenGrey. The systematic approach adopted in the search strategy is outlined in Table 1. In addition to electronic searches, manual searches were performed in leading endodontic journals to identify relevant articles that may not have been indexed in electronic databases, ensuring a more comprehensive literature review.

**Table 1:** Search strategy across various databases

Database	Search strategy	Number
PubMed	("post operative pain"[MeSH Terms] OR "irrigation" OR "laser-activated irrigation" OR "ultrasonic activated irrigation" AND ("root canal therapy") AND “conventional irrigation"[MeSH Terms] AND "diode laser" OR "necrosed teeth" OR "diode laser AND ultrasonics" OR "post-operative pain AND diode laser"[MeSH Terms] OR ("root canal therapy" AND "post-operative pain AND single visit endodontics" OR "post-operative pain AND ultrasonics" OR "laser AND single visit endodontics" OR "post operative pain" AND “randomized controlled trials” AND “comparative studies”	18
EBSCOhost	("post operative pain"[MeSH Terms] OR "irrigation" OR "laser-activated irrigation" OR "ultrasonic activated irrigation" AND ("root canal therapy") AND “conventional irrigation"[MeSH Terms] AND "diode laser" OR "necrosed teeth" OR "diode laser AND ultrasonics" OR "post-operative pain AND diode laser"[MeSH Terms] OR ("root canal therapy" AND "post-operative pain AND single visit endodontics" OR "post-operative pain AND ultrasonics" OR "laser AND single visit endodontics" OR "post operative pain" AND “randomized controlled trials” AND “comparative studies”	07
Google Scholar	("post operative pain"[MeSH Terms] OR "irrigation" OR "laser-activated irrigation" OR "ultrasonic activated irrigation" AND ("root canal therapy") AND “conventional irrigation"[MeSH Terms] AND "diode laser" OR "necrosed teeth" OR "diode laser AND ultrasonics" OR "post-operative pain AND diode laser"[MeSH Terms] OR ("root canal therapy" AND "post-operative pain AND single visit endodontics" OR "post-operative pain AND ultrasonics" OR "laser AND single visit endodontics" OR "post operative pain" AND “randomized controlled trials” AND “comparative studies”	426
Grey literature	("post operative pain"[MeSH Terms] OR "irrigation" OR "laser-activated irrigation" OR "ultrasonic activated irrigation" AND ("root canal therapy") AND “conventional irrigation"[MeSH Terms] AND "diode laser" OR "necrosed teeth" OR "diode laser AND ultrasonics" OR "post-operative pain AND diode laser"[MeSH Terms] OR ("root canal therapy" AND "post-operative pain AND single visit endodontics" OR "post-operative pain AND ultrasonics" OR "laser AND single visit endodontics" OR "post operative pain" AND “randomized controlled trials” AND “comparative studies”	05

Screening Process

The screening process was carried out in two phases. In the first phase, two independent reviewers (AG and KS) evaluated the titles and abstracts of all retrieved studies to exclude those that did not meet the eligibility criteria. Studies that fulfilled the inclusion criteria proceeded to the second phase, where the full texts were obtained and assessed for relevance. The same two reviewers independently screened the full-text articles, and any disagreements regarding eligibility were resolved through discussion. If consensus could not be reached, a third reviewer (AS) was involved to finalize the selection. To obtain additional clarifications or missing data, the corresponding authors of selected studies were contacted via email.

Data Extraction

Data extraction was conducted independently by two reviewers (AG and MR) to ensure accuracy and minimize bias. A pilot-tested, customized data extraction form was used in Microsoft Excel 2019 (Microsoft Corp., Redmond, WA, USA) to compile relevant study characteristics. The extracted data included details such as the author(s), country of study, year of study, sample size, study design, details about the activation systems used, outcome measures, key findings, and conclusions. The extracted data were cross-verified by both reviewers, and discrepancies were resolved through discussion. If disagreements persisted, a third reviewer (AS) was consulted to make the final decision.

Quality Assessment of Included Studies

The methodological quality of the included studies was evaluated using the Cochrane Collaboration Risk of Bias (ROB-2) tool [25]. The studies were assessed using a tool based on five domains, including randomization process, deviations from intended interventions, missing outcome data, measurement of the outcome, and selection of the reported result. Each study was assigned an overall risk rating based on these domains. A study was categorized as having a low overall risk of bias only if all domains had a low risk. If one or more domains were rated as high risk, the study was deemed to have a high overall risk of bias. Studies were assigned a moderate risk of bias when one or more domains were classified as uncertain, with none categorized as high risk.

Statistical Analysis

Using the random effects model, the meta-analyses were applied with RevMan 5.4 (RevMan 5.4, The Nordic Cochrane Centre, Copenhagen). Heterogeneity was assessed by a Q test and quantified with I^2^ statistics. Data on mean and standard deviation were obtained from selected studies. The postoperative pain score was considered the main outcome. In the studies with more than one laser intervention evaluated, all relevant experimental intervention groups of the study were combined into a single group. For analyses, if the test showed substantial heterogeneity (I^2^>50%), a random effects model was applied, or else (I^2^ ≤50%), a fixed effects model would be used. For the sake of providing only valid information, we have restricted reporting of the meta-analysis to only those intervals with heterogeneity of less than 50%.

Results

Study Selection

**Figure 1:**
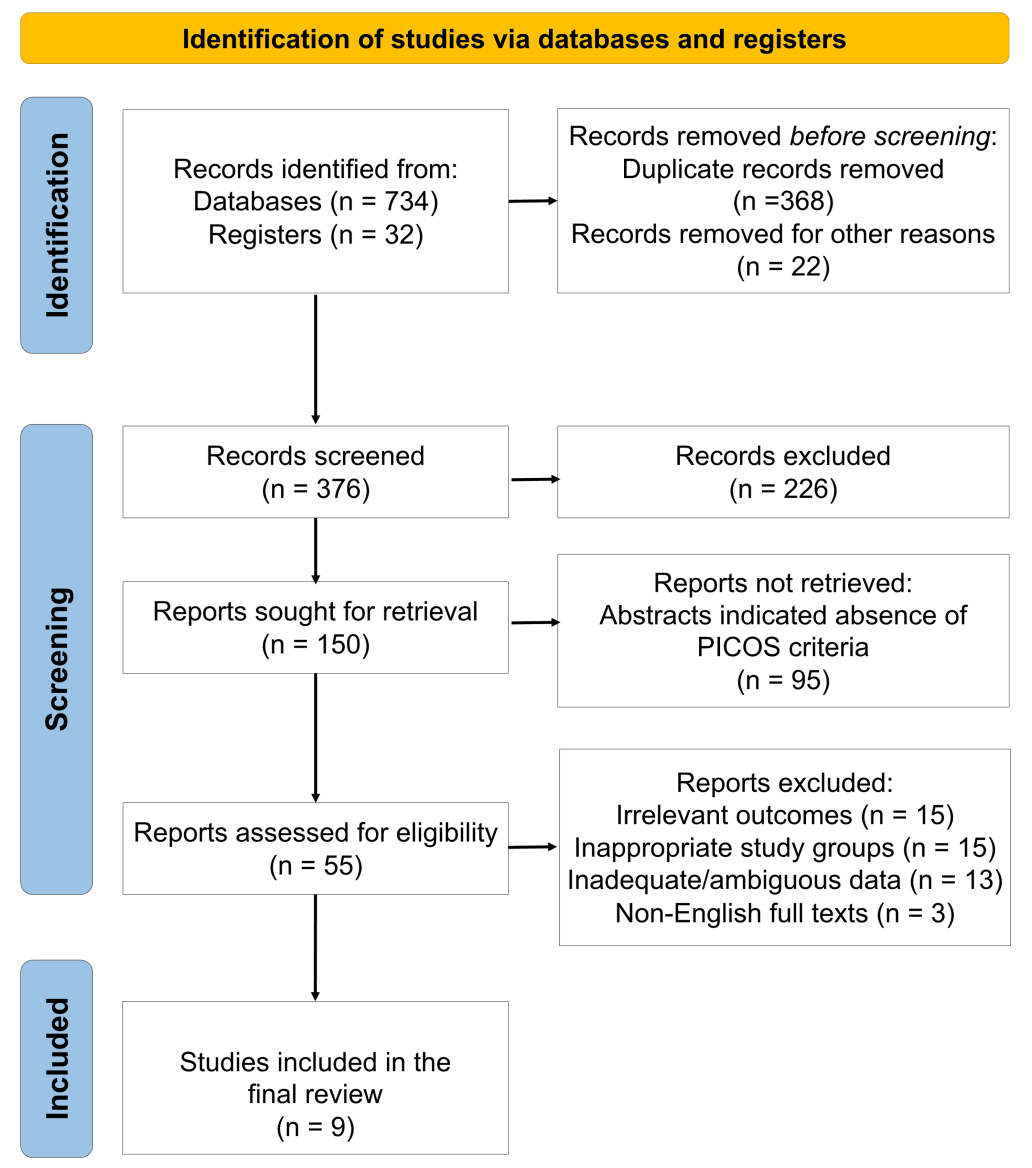
PRISMA flow diagram indicating the selection process of the articles in the present systematic review PRISMA: Preferred Reporting Items for Systematic Reviews and Meta-Analyses; PICOS: population, intervention, comparison, outcome, study design

The systematic search retrieved a total of nine randomized controlled trials satisfying the eligibility criteria [26-34]. The study selection process is illustrated in the PRISMA flow diagram (Figure 1), and the key characteristics of the included studies are presented in Table 2.

**Table 2:** Data extracted from all the studies included in the present systematic review LAI: laser-activated irrigation; PUI: passive ultrasonic irrigation; CUI: continuous ultrasonic irrigation; UAI: ultrasonic activated irrigation; PIPS: photon-induced photoacoustic streaming; SWEEPS: shock wave-enhanced emission photoacoustic streaming; CSI: conventional syringe irrigation: XP-Endo: XP-Endo finisher; LD: laser disinfection; LLLT: low-level laser therapy; IAS: irrigation activation system; NRS: numerical rating scale; VAS: visual analog scale; RCT: randomized controlled trial; CBCT-PAI: cone beam computed tomography periapical index; M: male, F: female

Author (year)	Study design	Country	Sample size	Age range (years)	Gender distribution	Diagnostic criteria	Intervention	Comparator	Outcome measures	Follow-up duration	Baseline pain score	Post-treatment pain score	Reduction in pain (%)	Statistical significance	Adverse events	Conclusion
Verma et al. (2020) [26]	RCT	India	69	18-60	Not specified	Chronic apical periodontitis with CBCT-PAI scores 3-5	LAI, PUI	CSI	Postoperative pain at 7 days (VAS score), Radiographic healing at 6 and 12 months (CBCT-PAI score)	12 months	Not specified	Significant reduction in CBCT-PAI scores in PUI and LAI groups compared to conventional syringe irrigation (p < 0.05)	Higher healing rates in LAI (42.1%) and PUI (36.8%) compared to syringe irrigation (10.5%)	Statistically significant reduction in CBCT-PAI scores in LAI vs. syringe irrigation (p < 0.05); No significant difference between PUI and LAI (p > 0.05)	No adverse effects reported	LAI and PUI demonstrated better clinical and radiographic healing compared to conventional syringe irrigation, with comparable success rates for PUI and LAI.
Dedania et al. (2021) [27]	RCT	India	44	18-60	M: 25, F: 19	Non-vital single-rooted teeth with fully formed apices	LD, PUI	Comparison between two irrigation techniques	Postoperative pain at 6, 24, 48 hours, and 7 days (Modified Verbal Rating Scale); Analgesic intake	7 days	Not specified	No significant difference in post-op pain at any time point (p = 0.086)	Pain reduction noted in both groups; slightly higher pain in laser disinfection group in the initial hours	No statistically significant difference (p = 0.086)	No adverse effects reported	Both PUI and laser disinfection were equally effective in reducing post-operative pain, though laser disinfection had a slight advantage in the initial hours.
Liapis et al. (2021) [28]	RCT	Belgium	56	18-78	M: 31, F: 25	Asymptomatic teeth requiring primary root canal treatment	UAI using Irrisafe tip, LAI using Er:YAG laser	Comparison between two irrigation techniques	Postoperative pain at 6, 24, 48, and 72 hours (VAS score); Analgesic intake	72 hours	Not specified	Significantly higher pain in UAI group at 6 hours compared to LAI (p < 0.05), no significant difference at 24, 48, and 72 hours	Pain reduced in both groups over time, no significant difference at later time points	Statistically significant pain difference at 6h (p < 0.05), but not at other time intervals	No severe adverse events; One patient in the UAI group reported severe pain but no complications	Both UAI and LAI resulted in low levels of postoperative pain, with LAI showing lower pain at 6 hours but no clinically significant difference at later time points.
Elmallawany et al. (2022) [29]	RCT	Egypt	40	20-45	Not specified	Necrotic mandibular molars requiring single-visit RCT	CUI, Diode Laser	CSI	Postoperative pain at 6, 12, 24, 36, 48 hours, and 7 days (VAS score); Bacterial reduction (CFU/mL)	7 days	Not specified	Significantly lower pain in CUI with diode laser group at all time points compared to conventional syringe irrigation (p < 0.001)	Higher bacterial reduction in CUI with diode laser (96.51%) compared to conventional syringe irrigation (79.96%)	Statistically significant difference in pain scores and bacterial reduction between groups (p < 0.05)	No severe adverse events reported	CUI with diode laser resulted in significantly lower postoperative pain and higher bacterial reduction compared to conventional syringe irrigation.
Abbara et al. (2023) [30]	RCT	Syria	60	25-44	M: 32, F: 28	Asymptomatic apical periodontitis in maxillary incisors	PUI, XP-Endo file, Diode Laser	Comparison among three irrigation activation systems	Postoperative pain at 1, 3, 7, and 14 days (VAS score)	14 days	Not specified	Diode laser group had significantly lower pain at 1, 3, and 7 days compared to PUI and XP-Endo Finisher (p < 0.05). XP-Endo Finisher group showed the highest pain at 1 and 3 days (p < 0.05). Pain disappeared in all groups after 14 days.	Pain decreased in all groups over time. Diode laser had the lowest pain, XP-Endo Finisher had the highest pain.	Statistically significant pain reduction in diode laser group compared to other groups at 1, 3, and 7 days (p < 0.05), but no difference at 14 days (p > 0.05).	No severe adverse events reported	Diode laser resulted in the lowest postoperative pain, XP-Endo Finisher caused the highest pain levels in the first 3 days, but pain disappeared in all groups after 2 weeks.
Krishnakumar et al. (2023) [31]	RCT	India	63	>18	Not specified	Symptomatic necrotic single-rooted teeth requiring primary endodontic treatment	CUI, LAI, Laser Irradiation	Comparison among three irrigation activation techniques	Postoperative pain at 24, 48 hours, and 7 days (VAS, NRS pain scales); Analgesic intake	7 days	Not specified	No significant difference in post-op pain among the three groups at any time interval (p > 0.05)	Pain reduced in all groups over time, with no statistically significant difference between CUI, LAI, and LI	No statistically significant difference (p > 0.05)	One patient in the LI group required analgesics at 24 hours; no severe adverse events reported	There was no statistically significant difference between CUI, LAI, and LI in postoperative pain reduction at any time interval, indicating similar efficacy in managing post-op pain.
Mathevanan et al. (2023) [32]	RCT	India	75	18-65	M: 38, F: 37	Symptomatic irreversible pulpitis in mandibular first molars	PUI using IrriSafe™, LAI using Er:Cr:YSGG laser, Conventional Needle Irrigation	Comparison among three irrigation activation techniques	Postoperative pain at 6, 24, and 48 hours (VAS score); Analgesic intake	48 hours	≥5 cm on VAS scale	PUI had the lowest post-op pain, followed by LAI; CNI had the highest pain at all time intervals (p < 0.05)	Pain reduced over time in all groups, with PUI and LAI showing significantly lower pain than CNI	Statistically significant difference between PUI, LAI, and CNI at all time intervals (p < 0.05)	No severe adverse events reported; CNI group had higher analgesic intake compared to PUI and LAI	PUI and LAI demonstrated significantly lower postoperative pain compared to CNI; PUI resulted in the lowest analgesic consumption.
Mittal et al. (2023) [33]	RCT	India	60	18-44	M: 28, F: 32	Symptomatic irreversible pulpitis in maxillary and mandibular molars	PUI, PIPS, SWEEPS	Conventional Needle Irrigation	Postoperative pain at 24 and 48 hours (VAS score); Analgesic intake	48 hours	8.93 - 9.13 (VAS 0-10 scale)	SWEEPS group had the lowest post-op pain at all time points (p < 0.05); PIPS group had significantly lower pain than CNI; Ultrasonic activation was superior to CNI but less effective than PIPS and SWEEPS	SWEEPS group had the highest reduction in pain, followed by PIPS, then PUI, with CNI showing the least reduction	Statistically significant difference between SWEEPS, PIPS, and other groups at 24h and 48h (p < 0.05)	No severe adverse events reported	Laser-activated irrigation systems (SWEEPS and PIPS) resulted in significantly lower postoperative pain than conventional and ultrasonic activation methods, with SWEEPS being the most effective.
Abbara et al. (2024) [34]	RCT	Syria	80	25-44	M: 36, F: 44	Asymptomatic necrotic maxillary incisors with large-sized periapical lesions	Diode Laser used as IAS, LLLT, Combination of IAS and LLLT	No laser application	Postoperative pain at 1, 3, 7, and 14 days (VAS score); Analgesic intake	14 days	Not specified	Group 4 (IAS + LLLT) had the lowest post-op pain, while the control group had the highest pain at all time points (p < 0.05)	Pain reduced in all groups over time, with the lowest pain levels in the combination group	Statistically significant differences between all groups at 1, 3, 7, and 14 days (p < 0.05)	No severe adverse events reported	The combination of diode laser IAS and LLLT significantly reduced postoperative pain compared to IAS or LLLT alone, suggesting its effectiveness in managing pain following endodontic treatment.

Study Characteristics

The included studies were conducted across four different countries, reflecting varied clinical protocols and population characteristics. The majority of the studies were from India (n=5), followed by Syria (n=2), Egypt (n=1), and Belgium (n=1). The total sample size across all nine studies was 547 patients. Most participants were between 18 and 65 years of age, although some studies extended the upper age limit up to 78 years. Gender distribution was reported in most studies, with a relatively balanced male-to-female ratio.

The diagnostic criteria used in the included trials primarily encompassed symptomatic irreversible pulpitis, necrotic pulpal conditions, and asymptomatic apical periodontitis, all of which required single-visit root canal treatment. The irrigation systems evaluated included diode lasers, Er:YAG lasers, SWEEPS, and PIPS techniques under LAI, as well as passive and continuous UI systems. Some studies also included conventional needle irrigation (CNI) and sonic activation for additional comparison.

Baseline and Post-treatment Pain Scores

Most studies used the Visual Analog Scale (VAS) or Numerical Rating Scale (NRS) to assess pain, although specific baseline scores were not consistently reported across all studies. Only Dedania et al. (2021) utilized the Modified Verbal Rating Scale for the assessment of pain in their study [27].

Across the nine included randomized controlled trials, baseline pain scores ranged from 5.96 to 9.42 on 10-point VAS or NRS, with the highest preoperative pain seen in the study by Mittal et al., 2023 on symptomatic irreversible pulpitis and apical periodontitis (9.13 on VAS scale) [33]. In contrast, asymptomatic cases, such as those in Liapis et al. (2021), reported baseline scores below 4 on the VAS [28]. Postoperative scores at six hours showed considerable differences between groups. Liapis et al. reported 4.9 VAS in the LAI group vs. 13.9 VAS in the ultrasonic group [28]. Mathevanan et al. (2023) found six-hour scores of 4.28 VAS for LAI, 4.00 VAS for PUI, and 5.60 VAS for CNI [32]. In Mittal et al. (2023), 24-hour pain scores were lowest in the SWEEPS group (0.93 VAS), followed by PIPS (2.00 VAS), with the CNI group reporting the highest pain (4.53 VAS) [33].

At 48 hours, LAI groups consistently showed lower scores (e.g., 1.20 VAS in Mathevanan et al., 0.13 VAS in the SWEEPS group of Mittal et al.) compared to UI or CNI [32,33]. Elmallawany et al. (2022) reported 5.1 VAS at 48 hours and 0.6 VAS at 7 days for laser-assisted CUI, significantly lower than syringe irrigation (28.6 VAS at 48 hours, 1.7 at seven days). By day 7, most groups reported minimal or no pain [29]. Abbara et al. (2023) found mean scores of 0.67 VAS in the diode laser group and 0.80 VAS in the PUI group [30], while Abbara et al. (2024) observed complete pain resolution (0.00 VAS) in the group receiving combined LAI and LLLT [34].

Overall, the trends indicate that patients presenting with symptomatic pulpitis or apical periodontitis had higher baseline pain levels. Post-treatment pain scores revealed significant variation depending on the activation method. In general, LAI showed lower pain levels within the first 24 to 48 hours compared to UI. However, by the end of the follow-up period (ranging from 48 hours to 14 days), both groups showed a comparable decrease in pain scores. Among the laser methods, diode lasers and SWEEPS techniques consistently demonstrated the most substantial reduction in postoperative pain. Ultrasonic methods also resulted in significant pain reduction compared to conventional irrigation techniques, albeit with a slightly delayed onset.

Statistical Significance in Pain Reduction

Statistically significant reductions in postoperative pain were reported in seven of the nine included studies (p < 0.05). Studies using diode lasers, SWEEPS, and PIPS recorded pain reductions exceeding 70% within 48 hours, while ultrasonic techniques generally showed a 50-65% reduction in pain during the same interval. Studies comparing multiple methods noted that while laser techniques offered quicker pain relief, the long-term pain scores equalized between UI and LAI by the end of the follow-up.

Effectiveness of Irrigation Techniques

In studies directly comparing LAI and UI, the former demonstrated faster pain relief within the first 24 hours post-treatment, particularly with diode lasers and photoacoustic streaming techniques. However, UI methods showed pain reduction comparable to LAI after 48 hours and were similarly effective by the end of the observation period. SWEEPS and PIPS showed enhanced effectiveness over both CNI and standard laser methods in one study. Overall, laser and ultrasonic methods were both superior to conventional syringe irrigation in reducing postoperative pain.

Adverse Events and Safety

No severe adverse events were reported in any of the included studies. One study recorded a single instance of severe pain in the ultrasonic group, which resolved without complications. Higher analgesic consumption was occasionally observed in groups receiving manual dynamic agitation or conventional irrigation, but this did not affect the comparative outcomes for LAI and UI.

Summary of Findings

Overall, both laser-activated and ultrasonic irrigation techniques were found to be effective in managing postoperative pain following single-visit endodontic treatment. Laser-based techniques, especially diode lasers and SWEEPS, showed faster pain reduction within the first 24 to 48 hours, while ultrasonic irrigation provided comparable long-term relief. These findings affirm the clinical utility of both methods as superior alternatives to conventional syringe irrigation for enhancing patient comfort and treatment outcomes.

Risk of Bias Assessment

**Figure 2:**
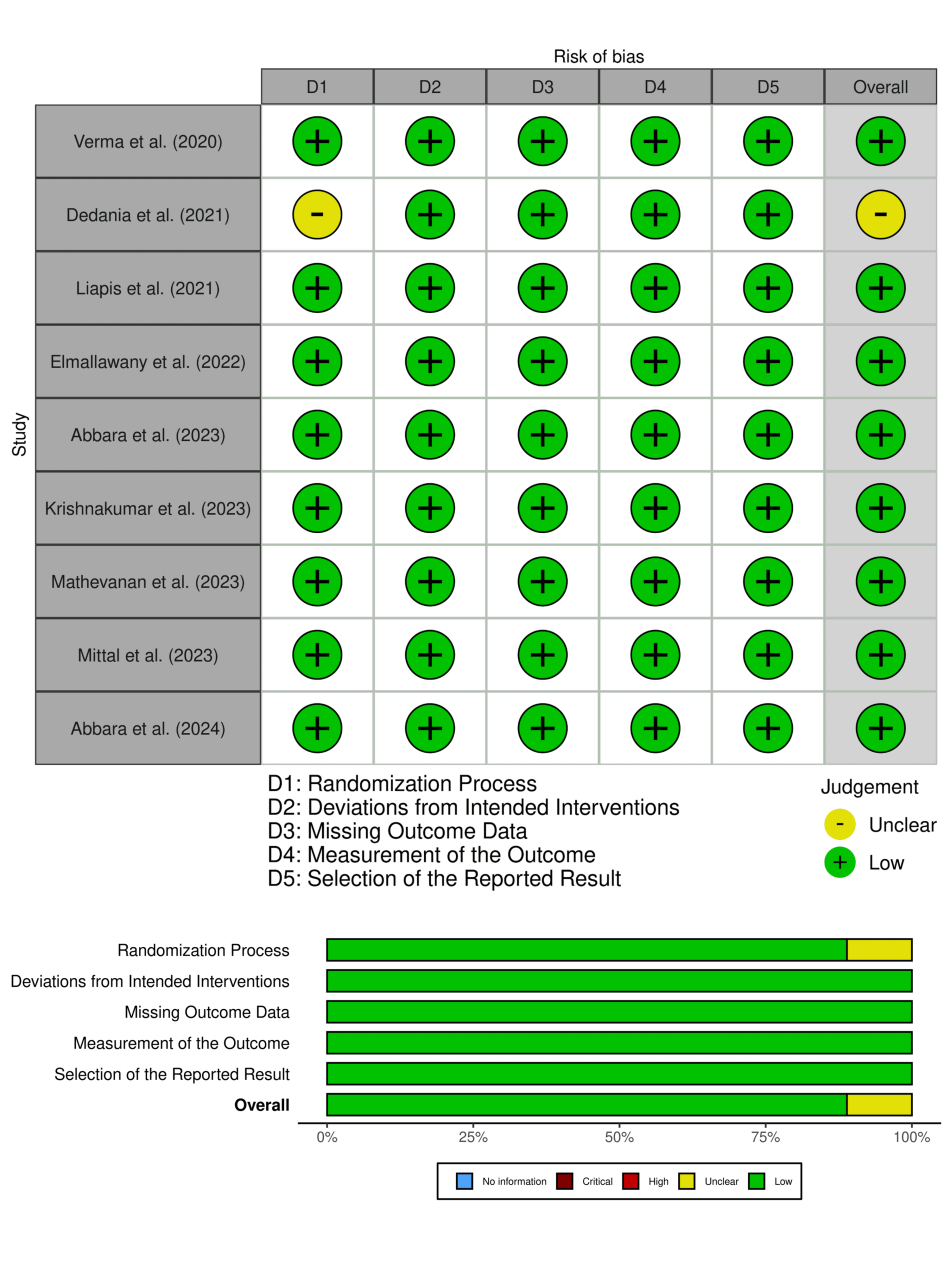
Risk of bias (RoB) in the included studies according to Cochrane RoB-2 tool References: [26-34]

The quality of the included randomized controlled trials was assessed using the Cochrane Risk of Bias 2 (RoB-2) tool across five domains. Of the nine studies included in this review, eight were judged to have a low overall risk of bias, indicating strong methodology and reliable outcome reporting. One study (Dedania et al., 2021) was assessed as having some concerns in the domain of randomization, primarily due to insufficient information regarding allocation concealment; however, it demonstrated low bias across all remaining domains [27]. All studies effectively minimized deviations from intended interventions, maintained complete outcome data with minimal attrition, and consistently utilized validated outcome measures such as VAS or NRS. The results were reported as per pre-established protocols, minimizing the likelihood of selective reporting. Overall, the included studies demonstrated a generally low risk of bias, supporting the internal validity and credibility of the synthesized findings (Figure 2).

Comparison of Postoperative Pain After 24 Hours

**Figure 3:**
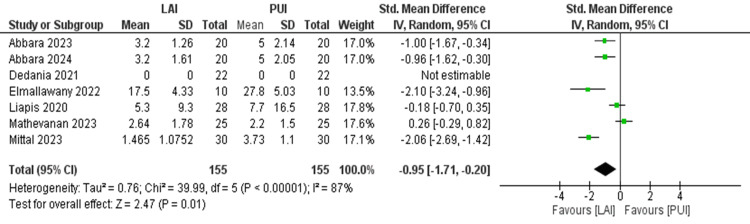
Forest plot showing statistical comparison of postoperative pain after 24 hours across the included studies

The meta-analysis was performed on seven studies that qualified with the required data outcomes that could be analyzed quantitatively. The results of the overall comparison have been depicted as a forest plot (Figure 3). With the meta-analysis conducted for selected studies, heterogeneity was more than 50% (I2 = 87%); hence, a random effect model was applied. The cumulative mean pain score after 24 hours was lower in the laser-assisted irrigation group as compared to the passive ultrasonic irrigation group with a standardized mean difference of -0.95 (95% CI = -1.71 to -0.20; Z value = 2.47), and the difference between the two groups was statistically significant (p=0.01).

Comparison of Postoperative Pain After 72 Hours

**Figure 4:**
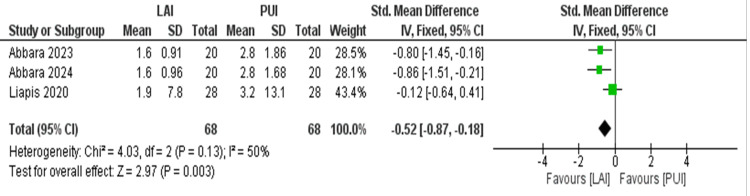
Forest plot showing statistical comparison of post-operative pain after 72 hours across the included studies

The meta-analysis was performed on three studies that qualified with the required data outcomes that could be analyzed quantitatively. The results of the overall comparison have been depicted as a forest plot (Figure 4). With the meta-analysis conducted for selected studies, heterogeneity was equal to 50% (I2 = 50%); hence, a fixed effect model was applied. The cumulative mean pain score after 72 hours was lower in the laser-assisted irrigation group as compared to the passive ultrasonic irrigation group with a standardized mean difference of -0.52 (95% CI = -0.87 to -0.18; Z value = 2.97), and the difference between the two groups was statistically significant (p=0.003).

Discussion

The findings of this systematic review highlight the significant role of advanced irrigation activation techniques, particularly LAI and UI, in reducing postoperative pain following single-visit root canal treatment. Across the included studies, both techniques demonstrated superior pain relief compared to conventional syringe irrigation, with laser-based methods showing a faster initial reduction in pain, while ultrasonic activation exhibited consistent and sustained pain relief over time. These outcomes are in corroboration with the fundamental principles of endodontic pain management, which emphasize the importance of efficient bacterial eradication, debris removal, and minimal apical extrusion of irrigants [35].

Pain Relief in the Initial Hours

One of the key observations was that LAI led to a more rapid reduction in pain, particularly within the first 24 to 48 hours post-treatment. Among the different laser types, diode and Er:YAG lasers demonstrated the most effective pain relief, likely due to their ability to induce significant acoustic microstreaming while also exhibiting photothermal and photobiomodulatory effects. The latter may play a crucial role in modulating inflammatory pathways, reducing nociceptor activation, and promoting faster tissue healing, ultimately leading to a quicker decline in postoperative pain perception [36].

In contrast, UI exhibited comparable pain reduction but with a slightly delayed effect compared to LAI. Passive UI and continuous UI were particularly effective in reducing postoperative discomfort, likely due to their ability to improve fluid penetration into complex canal anatomy. However, unlike LAI, which induces bactericidal effects through light energy, UI relies entirely on mechanical activation. This distinction might explain why studies assessing pain at very early time intervals (six to 12 hours) reported lower scores in LAI groups compared to UI groups. The absence of direct antimicrobial effects in ultrasonic techniques could result in a shorter delay in bacterial regrowth, which, although transient, could account for slightly prolonged inflammatory responses. In the meta-analysis, it was observed that the post-operative pain levels were significantly lower for LAI as compared to UI at 24-hour and 72-hour intervals.

Long-term Pain Relief

Despite these differences in initial pain reduction, the studies reported that by seven to 14 days postoperatively, both LAI and UI techniques exhibited similar pain levels. This suggests that while LAI accelerates initial pain relief, UI is equally effective in long-term pain reduction. This finding has important clinical implications, as it indicates that for practitioners seeking immediate pain relief, LAI may be the preferred choice. On the other hand, for clinicians who prioritize cost-effectiveness and ease of implementation, UI remains a highly viable alternative. Moreover, the lack of significant differences in pain reduction at later time intervals implies that the overall success of endodontic pain management is ultimately linked to adequate canal disinfection, regardless of the specific activation method employed.

Clinical Implications

The clinical implications of these findings are particularly relevant for managing patient expectations and optimizing treatment protocols. Patients undergoing root canal treatment often experience significant anxiety regarding postoperative discomfort, and these findings suggest that utilizing advanced activation techniques can substantially improve their experience [37]. Since pain perception is influenced by multiple factors, including tissue trauma, bacterial load, and immune response, selecting an appropriate irrigation method tailored to the individual case could enhance patient satisfaction and compliance [38]. For example, in cases with high bacterial burden, extensive periapical pathology, or symptomatic irreversible pulpitis, incorporating laser-activated irrigation may help achieve faster symptom relief. Conversely, in cases where cost and accessibility are concerns, ultrasonic activation provides an effective and more affordable alternative while still delivering significant improvements in patient comfort compared to conventional syringe irrigation.

One of the major drawbacks of conventional syringe irrigation is the inability to control irrigant flow beyond the apex, often leading to chemical irritation and periapical inflammation. Both LAI and UI reduce the risk of apical extrusion due to their efficient intracanal fluid dynamics, but studies suggest that UI may pose a slightly higher risk of extrusion, especially when high-powered activation is used. This could be due to the continuous movement of irrigants within the canal system in UI, whereas LAI relies on intermittent pulses of energy that create localized cavitation without generating excessive fluid pressure at the apex [39]. This finding reinforces the importance of proper technique selection based on case-specific factors such as canal morphology, periapical status, and the presence of open apices, where apical extrusion may be more problematic.

Furthermore, Mittal et al. (2023) assessed photoacoustic and photoactivated streaming techniques such as SWEEPS and PIPS and found that they achieved even greater pain reduction compared to conventional laser activation methods [33]. These techniques take advantage of shock wave generation and enhanced fluid energy transfer, leading to superior smear layer and debris removal while minimizing procedural discomfort [24]. Their impact on postoperative pain highlights the potential future directions for endodontic irrigation, where combining laser-induced streaming with optimized irrigant formulations could further enhance pain control and healing outcomes.

From a broader perspective, the absence of severe adverse events across all included studies underscores the safety and reliability of both ultrasonic and laser-based irrigation systems. While some studies reported higher analgesic consumption in groups with manual dynamic agitation, this likely reflects higher mechanical stress and inadequate fluid activation rather than any intrinsic issue with UI or LAI [30]. This reinforces the clinical preference for technologically advanced activation methods, as they not only enhance treatment efficacy but also improve patient comfort without introducing additional risks. The clear benefits in pain reduction, bacterial elimination, and enhanced healing make both LAI and UI valuable additions to the clinician’s armamentarium. However, the decision to use laser or ultrasonic activation should be based on case-specific factors, operator experience, and available resources. Future research should focus on long-term clinical outcomes, patient-reported quality-of-life measures, and comparative cost-benefit analyses to further refine the optimal irrigation protocols for modern endodontic therapy.

## Conclusions

The findings of this systematic review confirm that both laser-activated irrigation LAI and UI significantly reduce postoperative pain following single-visit root canal treatment, with LAI providing faster initial pain relief and UI demonstrating sustained long-term effectiveness. Laser techniques, particularly diode and Er:YAG lasers, as well as advanced streaming methods like SWEEPS and PIPS, showed the most substantial reduction in pain within the first 24 to 48 hours, while PUI and CUI remained highly effective alternatives with comparable pain reduction beyond a week. Both techniques outperformed conventional syringe irrigation, reinforcing their necessity in modern endodontic practice. No severe adverse effects were reported, further validating their clinical safety and efficacy. Given these results, the integration of LAI or UI should be considered a standard approach for enhancing patient comfort and improving endodontic outcomes, with selection based on case complexity, resource availability, and operator preference.
